# Evaluation in an emergency department of rapid separator tubes containing thrombin for serum preparation prior to hs-cTnT and CK-MB analyses

**DOI:** 10.1186/1472-6890-13-20

**Published:** 2013-06-13

**Authors:** Yasemin U Budak, Kagan Huysal, Mehtap Bulut, Murat Polat

**Affiliations:** 1 Department of Clinical Laboratory, Sevket Yilmaz Education and Research Hospital, Sevket Yilmaz Devlet Hastanesi. Biyokimya Laboratuari. Yildirim, Bursa, Turkey; 2 Department of Clinical Laboratory, Yüksek İhtisas Education and Research Hospital, Bursa, Turkey; 3 Department of Emergency Medicine, Sevket Yilmaz Education and Research Hospital, Bursa, Turkey; 4 Department of General Surgery, Sevket Yilmaz Education and Training Hospital, Bursa, Turkey

**Keywords:** Rapid serum tubes, Cardiac troponin T, Creatine kinase-MB

## Abstract

**Background:**

In our emergency department, we collect blood in Rapid Serum Tubes (RSTs; Becton Dickinson, Franklin Lakes, NJ), in which clotting times are reduced. We investigated the influence of RST use on cardiac troponin T (hs-cTnT) and creatine kinase-MB (CK-MB) test results, in comparison with the use of tubes featuring a separator gel containing a clotting activator (SSTs; Green-vac, Yongin, Korea).

**Methods:**

Samples from 60 patients were divided into equal aliquots and placed into RSTs and SSTs; hs-cTnT and CK-MB concentrations were determined using an autoanalyzer (Elecsys 2010) running commercial assays (Roche Diagnostics, Penzberg, Germany). Between-tube differences in CK-MB and hs-cTnT values were compared using the paired *t*-test, and correlations among variables were evaluated by calculation of Spearman correlation coefficients (r values). Deming regression analysis was performed and Bland-Altman plots were constructed.

**Results:**

The hs-cTnT and CK-MB test results obtained from samples placed into RSTs and SSTs did not differ (p > 0.1). The correlations between the concentrations of hs-cTnT and CK-MB in samples placed into RSTs and SSTs were good; both r values were unity (p < 0.001). Deming regression analysis yielded the equation: RST [hs-cTnT] = 0.98 SST [hs-cTnT] + 0.69 pg/ml; and RST [CK-MB] = 0.95 SST [CK-MB]–0.09 ng/ml. The biases of 1.4 pg/ml (95% CI: minus 8.1–10.7 pg/ml) for hs-cTnT levels and 0.249 ng/ml (95% CI: minus 0.682–1.681 ng/ml) for CK-MB levels assayed using either tube was acceptable.

**Conclusion:**

The hs-cTnT and CK-MB test results did not significantly differ when either tube was used. RST tube use was associated with a short clotting time; this was an advantage in an emergency laboratory setting.

## Background

Assay of total creatine kinase (total CK), CK-MB, myoglobin, and troponins are conducted on the sera of patients with suspected acute myocardial infarction (AMI) [1]. Cardiac troponins (cTn) are structural proteins unique to the heart. Detection of cTn in peripheral blood indicates cardiomyocyte necrosis [1]. A very sensitive assay for high-sensitivity cardiac troponin T (hs-cTnT) was recently developed, enabling measurements of concentrations 10-fold lower than previously. Use of this assay in patients with suspected acute coronary syndromes has improved the accuracy of MI diagnosis compared with the standard cTnT assay [2-5]. In an emergency department, it is important that the concentrations of cardiac markers be determined rapidly after sample-taking; hence short “turnaround times” (TATs) are required. Existing guidelines recommend that cardiac biomarker TATs should be < 60 min [6].

Rapid technological advances (yielding “Stat” tests) have dramatically improved the TAT. Stat tests are optimal when preanalytical delay is also reduced. Thus, preanalytical processes should be carefully planned and workup time minimized.

Blood specimens collected in plasma tubes do not clot prior to centrifugation. This allows blood to be drawn, mixed, and immediately centrifuged, yielding rapid test results. However, analyte stability is greater in serum than in plasma [7,8], and differences between plasma and serum test results have often been documented [9]. Recently, Gerhardt et al. [10] and Stiegler et al. [11] showed that the levels of cardiac troponin T (cTnT) were 15% lower in heparinized plasma than in serum. Stiegler [11] suggested that the difference could be explained by conformational changes of the antigen induced by direct interactions between the positively charged (pI 5.1) cTnT molecule and negatively charged heparin. Such complexes can interfere with antibody-antigen interaction, as shown previously by Katrukha et al. [12]. On the basis of these results, Roche Diagnostics, manufacturer of the cTnT assay, have advised customers not to use heparinized plasma for assay of cTnT (3rd generation Elecsys® TnT assay).

Over the past few years we have used SSTs for blood collection, as the tubes facilitate rapid separation of serum from cells [13]. SSTs contain a gel barrier that moves to the serum/clot interface during centrifugation [13]. The RST is a recently released serum separator tube containing thrombin as a clotting activator, affording rapid serum separation and short test TATs [14], both of which are invaluable in emergency situations.

If a switch to use of RST tubes is contemplated to reduce TATs, it is important to compare test values obtained using different tubes. We assessed the comparability of hs-cTnT (Roche Diagnostics, Germany; lot161334) and CK-MB (Roche Diagnostics, Germany; lot161515) test results from patients whom we suspected had experienced acute myocardial infarction (AMI) using the new RST tubes (BD, Franklin Lakes NJ; lot 110408) and previously employed SSTs (Green-vac, Yongin, Korea; lot 110218) for specimen collection. Data were obtained using a Roche Diagnostics Elecsys 2010 analyzer (Roche Diagnostics, Penzberg, Germany) running commercially available assays.

## Methods

This study was approved by Sevket Yilmaz Research and Education Hospital Ethics Committee. The study was conducted over a period of 10 days and included data from 60 patients.

Patients admitted to our emergency department with a chief complaint of chest pain and with a request for CK-MB and hs-cTnT analysis, regardless of age or gender, were included. Blood samples were drawn by trained technologists and collected into RST and SST collection tubes in random order, with the tubes filled to capacity. In line with the manufacturers’ instructions, RSTs were inverted 5–6 times and centrifuged (1600 × g, 10 min) after 5 min whereas SSTs were inverted 5–6 times and centrifuged after 30 min (1600 × g, 10 min). Serum CK-MB (mass) and hs-cTnT concentrations were measured using commercially available electrochemiluminescence immunoassays on an Elecsys 2010 analyzer (Roche Diagnostics GmbH, Mannheim, Germany), with each sample tested in duplicate.

Prior to specimen analysis, we ran a two-level quality-control (QC) test using materials supplied by the tube manufacturers (PCTN1 lot 164408 and PCTN2 lot 163162). Between-day differences in QC sample results were determined after analysis, in duplicate, on each of 20 days, using a single reagent batch and a single calibration curve. Within-day precision was calculated by running 20 replicates simultaneously.

### Statistical analysis

Data were evaluated using SPSS version 13.0 (SPSS Inc., Chicago, IL) and Analyse-It version 2.04 (Analyse-It Software, Leeds, UK). Descriptive statistical values (arithmetical and geometric means, with SDs) were calculated in a conventional manner. Differences between CK-MB and hs-cTnT values of samples obtained from RSTs and SSTs were compared using the paired *t*-test. Correlations were determined via calculation of Spearman correlation coefficients (r values). For all statistical comparisons, p values less than 0.05 were considered significant. Data concordance was evaluated via Deming regression analysis. The mean of differences (bias) and limits of agreement were calculated using the Bland and Altman method [15].

## Results

For quality assurance purposes, our laboratory participates in an external quality assessment scheme run by Labquality, Helsinki, Finland. At the time of the present study, the QC program reported average values obtained in 107 participating laboratories. For CK-MB, the mean was 4.6 μg/l and our value was 4.7 μg/l; for CK-MB, the mean was 2.7 μg/l and our value was 2.9 μg/l; for hs-TNT, the mean was 882.7 ng/l and our value was 952.7 ng/l; and for hs-TNT the mean was 1,337 ng/l and our value was 1,348.7 ng/l. All of our data were within ±2 SDs of the mean values.

Intra- and inter-day variabilities were determined by multiple assays of QC samples, within day and between day CVs were < 6% for both assays. For QC1, the mean CK-MB was 6.2 μg/l and the mean hs-cTNT was 28 ng/l; for QC2, the means were 49.6 μg/l and 156 ng/l, respectively.

The hs-cTnT values of samples analyzed in the present study ranged from 3–712 pg/ml and the CK-MB values from 1.03–75.83 ng/ml. The mean CK-MB level obtained using RSTs was 6.56 ± 13.12 ng/ml whereas that for SST samples was 6.81 ± 13.58 ng/ml (paired *t*-test: 0.002). The mean hs-cTnT value obtained using SST tubes was 67.3 ± 7.3 pg/ml and that for samples placed into RSTs 73.5 ± 7.9 pg/ml.

**Figure 1:**
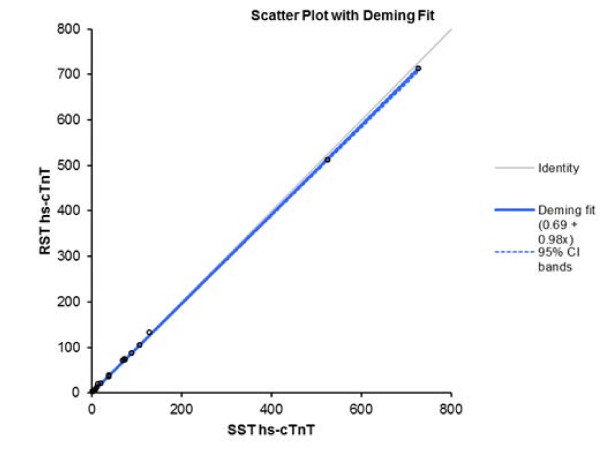
**Deming fit (solid black line) with 95% confidence intervals for the SST hs-cTnT results vs RST results.** Concentrations are expressed as picograms per milliliter.

**Figure 2:**
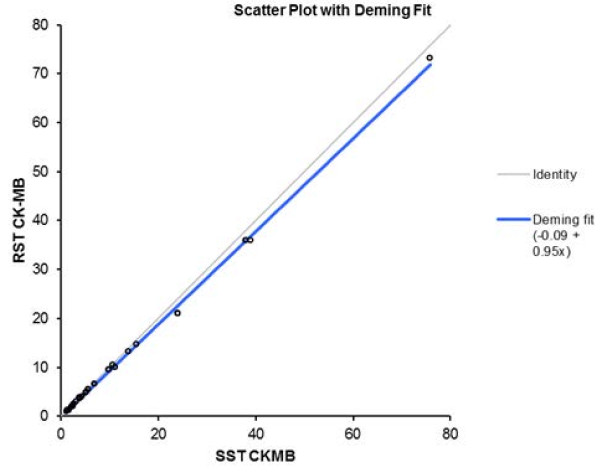
**Deming fit (solid black line) with 95% confidence intervals for the SST CK-MB results vs RST results.** Concentrations are expressed as nanograms per milliliter.

**Figure 3:**
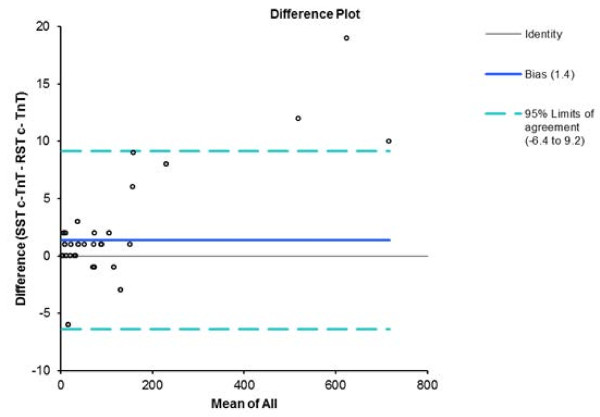
Bland-Altman plot of SST and RST tube hs-cTnT results showing the 95% limits of agreement.

**Figure 4:**
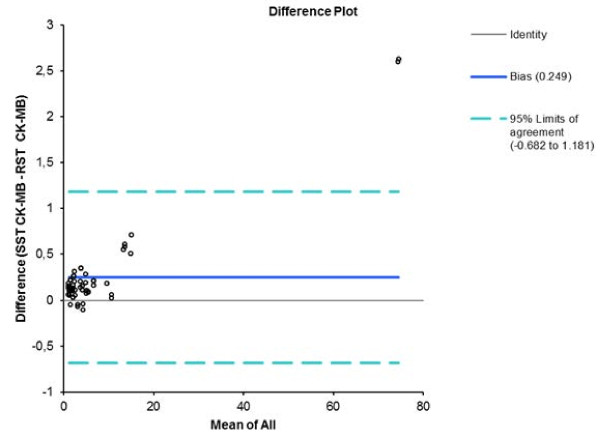
Bland-Altman plot of SST and RST tube CK-MB results showing the 95% limits of agreement.

The Spearman correlation coefficients were r = 1.0 (p < 0.001) when hs-cTnT and CK-MB assay data were compared. The correlations between hs-cTnT and CK-MB serum concentrations measured using RSTs and SSTs were very strong (r = 1.0 for both) (p < 0.001) (Figures 1 and 2). The bias values were 1.4 pg/ml (95% CI: minus 8.1–10.7 pg/ml) when the hs-cTnT values yielded upon use of either tube type were compared (Figure 3), and 0.249 ng/ml (95% CI: minus 0.682–1.681 ng/ml) for CK-MB (Figure 4). Deming regression analysis of hs-cTnT measurements obtained using either assay, on samples from all 60 patients (Figure 1), yielded the following data: slope, 0.98 [95% confidence interval (95% CI), 0.97–0.98]; and intercept 0.69 pg/ml (95% CI, minus 0.40–1.78 pg/ml). The RST hs-cTnT value was 0.98 × (the SST hs-cTnT value) + 0.69 pg/ml. Deming regression analysis of CK-MB levels yielded the following data: slope, 0.95 (95% CI, 0.90–1.00); and intercept, minus 0.09 ng/ml (95% CI, minus 0.51–0.32 ng/ml).

## Discussion

In the present study, we compared data from samples collected from patients undergoing clinical testing for suspected or confirmed MI, rather than those from samples obtained from healthy volunteers in a non-emergency environment. We found that the Spearman correlation coefficient was unity, indicating a perfect correlation, when hs-cTnT and CK-MB test results on samples collected into RSTs and SSTs were compared.

Clotting should continue for 30 min to ensure complete clot formation with SST tubes [10]. If a tube is centrifuged too soon, gelatinous and/or fibrin-containing serum samples will be obtained; these will clog the analyzer probes. Roberts et al. [16] observed that incompletely clotted specimen contributed to the false elevations in cardiac TnI concentrations with the Stratus II batch analyzer (Dade International). In emergency laboratories, this timeframe is often unrealistic. Complete clotting may occur in 5 min if blood sample tubes contain thrombin [14], the RSTs used in the present study are such tubes. We have shown herein that simultaneous quantification of CK-MB and hs-cTnT in samples derived from RSTs and SSTs yielded similar results.

In our present work, Deming regression analysis yielded the appropriate y-intercept value of zero for both tests but the slope of the regression line did not approximate unity, suggesting that, in addition to the presence of a small constant bias, a small (and nonsignificant) proportional difference existed between the test results obtained upon use of the two tube types [17].

The acceptable degree of imprecision of both the CK-MB and hs-cTnT assays is < 10% at the 99th percentile reference level [18]. In our hands, the imprecision levels of either assay were below this value. Notably, participation of our laboratory in internal and external quality assurance programs, and use of an automated analyzer, indicate that RST tubes may be used to reduce TAT. The quality of assay data is high. Importantly, many reports have shown that serum, rather than heparinized plasma, should be used for cardiac marker determination [10,11]. In a recent study, Strathmann et al. [19] showed that specimens collected into RSTs yielded fewer false-positive immunoassay results than did those collected as heparinized plasma samples when the Beckman Coulter Unicel DxI instrument was used to evaluate the levels of troponin I and the creatine kinase-MB isozyme.

The US CLIA 1988 rules indicate that differences of 7.8% in CK-MB levels, and 23% in cTnT levels, around target values, are acceptable [20] because of biological variation. In the present study, we observed that the hs-cTnT concentrations of serum samples collected into SSTs were slightly higher than were those of samples collected into RSTs. We also found that the serum CK-MB concentrations of samples collected into SSTs were somewhat higher than were those of samples collected into RSTs, but no detected difference was clinically significant. The bias values were smaller than are the US CLIA 1988 targets. The bias may be attributable to the fact that samples taken into SSTs have a longer clotting time; RST samples are analyzed about 30 min earlier than are SST samples. Also, the two tube types employ different clotting agents.

An increase of >2.0 ng/ml in CK-MB level compared to baseline concentrations is indicative of myocardial damage in patients without AMI. For both tube types, the CK-MB assay bias was 0.249 ng/ml, with zero included in the 95% confidence intervals. Such biases are acceptable [21], although additional data are needed to support the hypothesis that results from SST and RST are clinically interchangeable.

Heparin is utilized clinically to inhibit clotting in critical care patients. Blood samples from patients administered heparin prior to blood collection can contain excess concentrations of heparin, increasing clotting time in the collection tube and thereby increasing the potential for the formation of “latent” fibrin in the preanalytical phase. Although few of the samples in this study were from patients receiving heparin therapy, the inclusion of this subgroup may have confounded our results and constitutes a limitation of this study. Additional studies are needed to assess the influence of heparin therapy on CK-MB and hs-cTnT concentrations measured in RST and SST tubes.

Preanalytical variables associated with blood collection should be further standardized to ensure the accuracy of test results. It is impractical to expect tube manufacturers to test their tubes on all possible assay platforms; this is a task for individual laboratories.

## Conclusions

We conclude that RSTs are suitable for sample collection prior to CK-MB and hs-cTnT analysis in a hospital emergency laboratory. RST use is associated with a short clotting time, which is valuable in an emergency setting. Additionally, the tubes are inexpensive.

## Abbreviations

AMI: Acute myocardial infarction; cTn: Cardiac troponins; CK: Creatine kinase; hs-cTnT: High-sensitivity cardiac troponin T; RST: Rapid serum tubes; SST: Serum separator tubes; TAT: Turnaround times

## Competing interests

The authors declare that they have no competing interests.

## Authors’ contributions

YB was participated in the study design, the acquisition of data, helped to perform the statistical analysis, and drafted the manuscript. KH participated in the statistical design of the study, performed and made substantial contribution to the statistical analysis and interpretation of data. MP and MP were involved with contributing data and helped critically revise the manuscript. All authors read and approved the final manuscript.

## Pre-publication history

The pre-publication history for this paper can be accessed here:

http://www.biomedcentral.com/1472-6890/13/20/prepub
